# LabVIEW 2010 Computer Vision Platform Based Virtual Instrument and Its Application for Pitting Corrosion Study

**DOI:** 10.1155/2013/193230

**Published:** 2013-04-04

**Authors:** Rogelio Ramos, Roumen Zlatev, Benjamin Valdez, Margarita Stoytcheva, Mónica Carrillo, Juan-Francisco García

**Affiliations:** Engineering Institute, Autonomous University of Baja California, Boulevard Benito Juarez S/N, 21280 Mexicali, BCN, Mexico

## Abstract

A virtual instrumentation (VI) system called VI localized corrosion image analyzer (LCIA) based on LabVIEW 2010 was developed allowing rapid automatic and subjective error-free determination of the pits number on large sized corroded specimens. The VI LCIA controls synchronously the digital microscope image taking and its analysis, finally resulting in a map file containing the coordinates of the detected probable pits containing zones on the investigated specimen. The pits area, traverse length, and density are also determined by the VI using binary large objects (blobs) analysis. The resulting map file can be used further by a scanning vibrating electrode technique (SVET) system for rapid (one pass) “true/false” SVET check of the probable zones only passing through the pit's centers avoiding thus the entire specimen scan. A complete SVET scan over the already proved “true” zones could determine the corrosion rate in any of the zones.

## 1. Introduction

The increased application of self-constructed LabVIEW-based chemical virtual instruments (VIs) is due to their flexibility and ability to satisfy all the specific user requirements combined with the simplicity of the construction. Many configurations of LabVIEW-based VI have been reported until now corresponding to their specific chemical application defined by the user needs. Meng et al. [1] described a VI system based on LabVIEW 8.0 for ion analyzer which can measure and analyze ion concentrations in solution, comprising a high input impedance voltmeter (widely used in measuring the EM generated by ion selective electrode), a homemade conditioning circuit, data acquisition board, and a computer. It can calibrate automatically the slope, temperature, and positioning. When applied to determine the reaction rate constant by *pX*, it achieved live acquiring, real-time displaying, automatic processing of testing data, generating a report of results, and other functions. This method simplifies the experimental operation, avoids complicated procedures of manual data processing and personal error, and improves veracity and repeatability of the experiment results. Wang et al. [2] reported a LabVIEW-based chemical virtual instrument (VI) for temperatures and pressures measurement. By selecting hardware modules, such as the PCI-DAQ card or serial port method, and the software modules, different kinds of sensors can be used for creating different chemical instruments allowing extremely flexible solutions for automatic measurements in the physical chemistry research. Lenehan et al. [3] developed a LabVIEW-based software for the automation of a sequential injection analysis instrument for the determination of morphine. The detection is based on chemiluminescence reaction with acidic potassium permanganate in the presence of sodium polyphosphate. The authors reported excellent analytical characteristics of the developed LabVIEW-based software system to be achieved: the precision of the determination was 0.7% measured by the relative standard deviation for five replicate measurements of morphine standard, and the limit of detection was determined to be as low as 5 · 10^−11^ M. Bowie et al. [4] reported a flow-injection- (FI-) based instrument under LabVIEW control for iron monitoring in marine waters. The instrument incorporates a miniature, low-power photomultiplier tube (PMT), and a number of electric and solenoid actuated microvalves and peristaltic pumps. The software allows full control of all flow injection components and the processing of the data from PMT. The optimized system is capable of 20 injections per hour, including preconcentration and wash steps. The analytical characteristics of the developed LabVIEW-based system are reported to be as follows: a detection limit of 21 pM at linear range of 21–2000 pM with a 60-second sample load time and average precision between replicate FI peaks of 5.9 ± 3.2% (*n* = 4) over the linear range.

The flexibility of the VIs allows their application practically in any branch of the chemical technology. For example, the quality control of the conversion coating on aluminum alloys requires the determination of the pits number appearing on the 3 × 10 inches control specimens after their testing in saline chamber at extreme conditions: high temperature, high relative humidity, and high saline concentration, according to the standard ASTM B117. According to this standard, the number of the appearing pits is the measure of the corrosion resistance of the protective coating. The pits counting however actually made by simple specimen observation results in subjective errors due to the bad distinction of the pits from some “pits-like” simple stains and hence false results about the corrosion resistance of the conversion coatings. That is why a rapid and subjective error-free method for pits counting is necessary. Thus, the purpose of the present work is to develop such a VI and to test it on real specimens for express and objective pits counting.

Such a VI must determine the pit centers coordinates, pit areas, their traverse lengths, and the densities using blobs analysis resulting in a map file which can be used further by a SVET system [5–7] to perform a rapid (one pass) “true/false” check of the probable pit containing zones only, without scanning of the entire specimen. A VI fulfilling all these requirements using a particle analysis based on blobs analysis [4] included in IMAQ libraries [5] for LabVIEW 2010 called localized corrosion image analyzer (LCIA) was developed and described in the present paper. Its hardware and software are presented together with some application for pit recognition on real metal samples.

The specimen optical scan performed by a digital microscope connected to a PC yields a database file (a map file) containing the coordinates of all the surface defects similar to pits, not only the ones caused by corrosion. Image analysis performed by LabVIEW 2010 was applied for preliminary image recognition and distinction of the pits. The created map must be actualized by “true/false” SVET test application allowing the distinction of the true corroded (pit containing) zones from the “corroded-like” ones to be used further from a SVET system for corrosion rate determination. The true/false test is a rapid single linear SVET scan over the centers of the probable zones in which coordinates are saved in the created by the VI LCIA map file. The simple linear SVET scan will result in a specific and easy recognizable peaks contained curve in coordinates: current intensity/coordinate.

The scanning vibrating electrode technique (SVET) which was developed for biological applications [5] was adapted later by Isaacs [6, 7] for corrosion studies of pitting, intergranular, and galvanic corrosion. SVET allows the determination of the current distribution above the metal surface measuring the potential gradient (voltage drop) proportional to the ionic current density Δ*E* = (*I*_1_ − *I*_2_)*R* that appears between the two levels of the electrode vibration (from 1 to 100 *μ*m) above the studied surface. SVET is a further development of the scanning reference electrode technique (SRET) [8, 9] but provides higher sensitivity due to a.c. signal measurement allowing obtaining a higher signal to noise (*S*/*N*) ratio due to the better noise suppression. For example, the minimal voltage drops levels measurable by SRET are about 200 *μ*V [10, 11], while 5 *μ*V can be achieved by SVET [12–14].

The LabVIEW-based VI subject of the present paper employs the first main point of the approach developed by the authors based on the following two main points:

- computer vision application for video inspection by digital microscope of the surface of interest and database (map file) creation of the probable pits containing areas;
- rapid “true/false” test by single linear SVET scan for real pits distinction followed by a map file actualization.

Additionally, a complete SVET scanning of the recognized real pits areas can be performed if the corrosion rate determination is necessary. The VI performing the video inspection and the creation of the map file only is the subject of the present work, while the SVET VI is the subject of another publication.

## 2. System's Hardware Configuration

**Figure 1:**
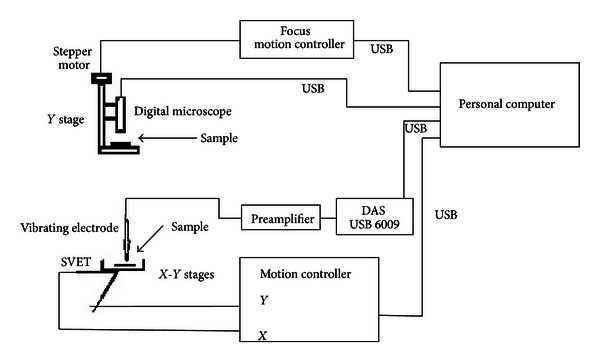
VI LCIA system configuration.

Three main devices were involved in the system hardware configuration: a video inspection mighty scope near infrared 10x–200x, 1.3 MP, 1/4′ CMOS color, 6 LED light type operating at 850 nm, USB 2.0 interfaced digital microscope with a homemade lineal stage focus controlling mechanism in the range from 8.5 mm to 112 mm, with stepper motor NPM PF35-24C1, a homemade SVET device with *X*-*Y *stages, stepper motors VEXTA model PX245 M-01A, and a personal computer Dell Optiplex 7010 Core i7 CPU @ 3.40 GHz, Windows 8 Pro operating system as illustrated in Figure 1.

The optical inspection by a digital microscope allows the capturing of full images from 4 mm² to 1700 mm² (optical zoom in the range from 10x to 200x), respectively, of the studied surface with a resolution of 1600 to 1200 effective pixels at shot speed user controllable from 1 to 1/1000 sec. The focus adjusting mechanism (*Y* stage, Figure 1) was driven by a stepper motor controlled by the LCIA which controls also the real time image capturing and its transfer through the USB interface. The white balance was carried out automatically employing the integrated 6 white LED light ring around the lenses. 

The homemade SVET device [15] which uses the map file produced by the VI subject of the present work consists of a vibrating electrode with edge diameter of approximately 1 *μ*m, 100 mV p-p, and frequency of the vibration of 60 Hz at amplitude of the displacement adjustable in the range between 1 and 100 *μ*m over the specimen surface. The vibrating electrode was mounted on *X*-*Y* linear stages mechanism providing 5 *μ*m/step resolution. The SVET electrode *X*-*Y* displacement and vibration were controlled by a separate VI instrument running its own software and communicating with the LCIA, using the map file containing the pits coordinates previously obtained with the LCIA. The SVET analog signal was amplified by a controlled gain high input impedance instrumentation preamplifier (10^13^ Ohm/100 fA), and after its digitalizing by national instruments, model USB 6009 data acquisition system (DAQ) digital signal was transferred to the PC through the USB port and processed by its VI lock-in amplifier LabVIEW tool. 

### 2.1. System's Operation

The LCIA focuses, captures the image of the specimen surface, and analyzes it creating a database file in real time containing the exact coordinates, area, and transverse length of probable pits. This file is employed by a SVET system with its own control software, performing the true/false test to distinguish the true pits coordinates. In “true” case, a complete SVET scan of the probable area can be performed and the corrosion rate be determined, if preliminary chosen by the operator in the interface screen. In “false” case, the SVET electrode goes to the next probable area up to the last using optimized trajectory. In both cases, after the true/false test performance, an updating of the already existing database file takes place.

## 3. Programming

**Figure 2:**
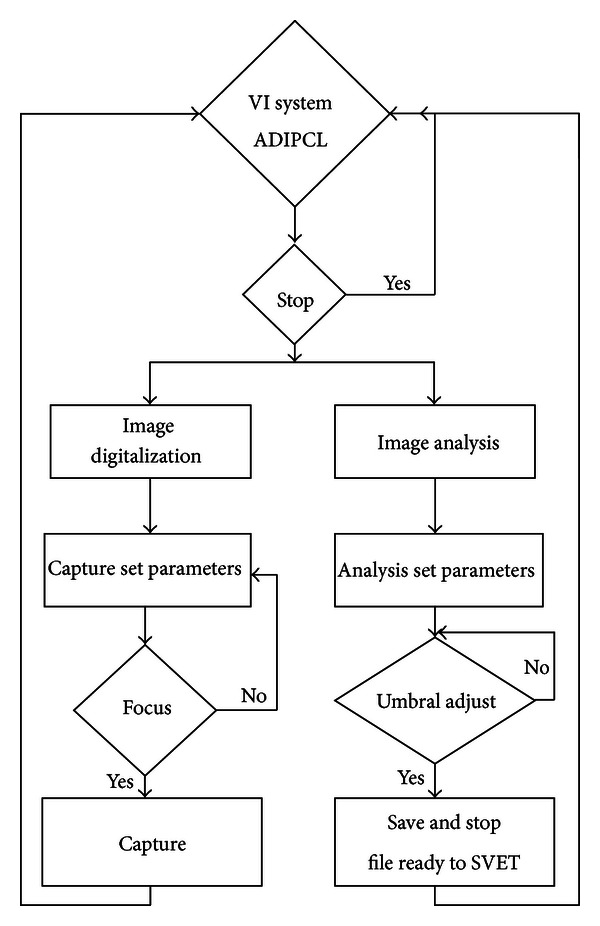
Flow diagram for VI LCIA.

The flow diagram of VI LCIA system shown in Figure 2 is divided in two main subvirtual instruments: *image digitizing sub-VI* and *image analyzing sub-VI,* both executed to get the complete pits location information. The *image digitizing sub-VI* creates the sample's digital image by making a preview, focusing, image capturing, histogram display, and focus verification entirely synchronized in real time with the *image analyzing sub-VI*. Both sub-VIs are implemented in “*stacked sequence 1*” structure where the *image digitizing sub-VI* is executed for *frame 0* of *stacked sequence 1* and the *image analysis sub-VI* is executed for *frame 1* of *stacked sequence 1* (see Figure 2).

### 3.1. Digitizing Sub-VI: General Description

**Figure 3:**
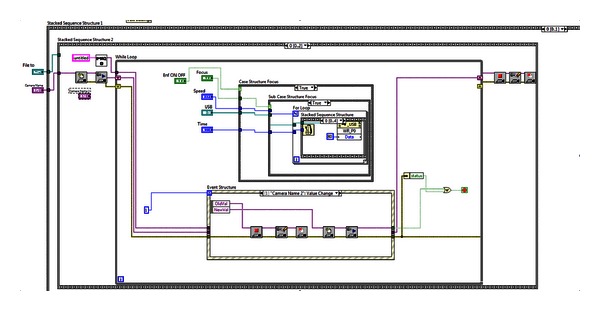
Digitizing sub-VI: image preview and focusing.

The *image digitizing sub-VI* is located in *frame* 0 of the *Stacked Sequence 2* inside the *Stacked Sequence 1 *shown in Figure 3, where the functions: focus and image preview, are performed simultaneously within a “while loop execution control.” The focus has two nested “case” structures. When the main “case” structure is true, the focus starts by the execution of “focus section” sub-VI through a “case true substructure”; this process calls up the node for writing via USB to control the driver of stepper motor mounted on the focus mechanism; it focuses by using a “for” cycle and a structure type *stacked sequence* (programming shown on Figure 3). When the main case structure is false, however, it calls up through the USB port the writing node which indicates the motor driver to de-energize the motor, and by this way, the focusing process stops. The previously mentioned procedure sets the forward or backward sequence to control the execution time, the speed of the focus mechanism stepper motor, and the focus termination through the control driver.

### 3.2. Digitizing Sub-VI: Image Preview and Real-Time Focusing

The image preview is made by using the LabVIEW Ni IMAQdx libraries, tools that allow the reading of camera or microscope through a USB port. This job is performed within an “event” structure (see Figure 3) and allows simultaneous real-time image observation and focusing.

The image preview is taken by the application of the LabVIEW Ni IMAQdx tool [16] allowing automatic detection and camera(s) selection by the user via the USB port. This sub-VI is implemented outside the “while loop” and inside the “event” structure shown in Figure 3.

### 3.3. Digitizing Sub-VI: Image Capture, Focus Verification, and Image Save

**Figure 4:**
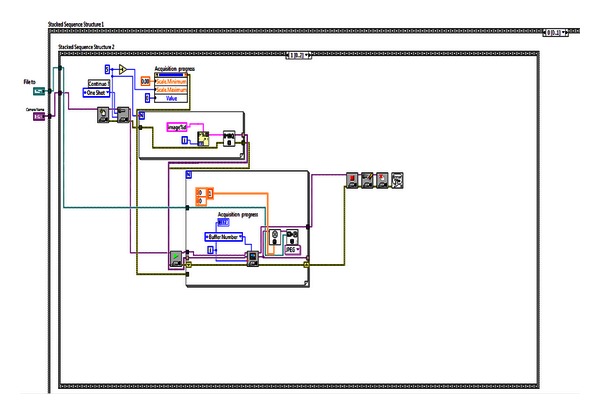
Sub-VI for image caption.

**Figure 5:**
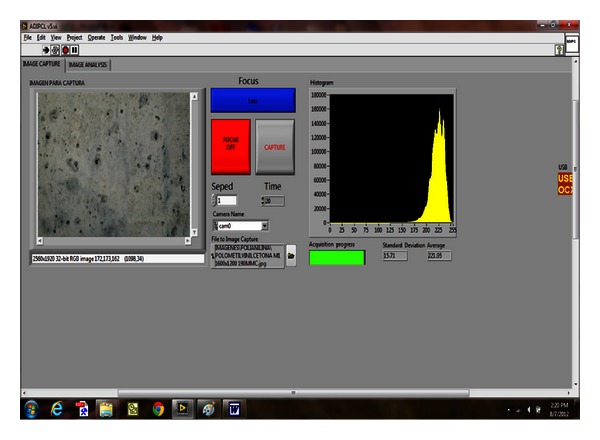
Image's caption front panel.

Once the “capture” button, located on the sub-VI front panel is pressed, the *frame 0* of stack sequence 2 terminates, and the *frame 2* starts automatically, capturing the image and saving it in *.jpg format file (the programming is shown in Figure 4). After saving the generated file the sub-VI *“content”* generates the corresponding histogram for the image capturing.  Figure 5 shows the user graphic interface (the front panel of VI LCIA for the image digitizing), where the corroded surface image already captured is displayed together with the corresponding histogram. According to the histogram values the user decides to keep the image for further analysis or to delete it and run the process again.

### 3.4. Image Analysis Sub-VI

In the image analysis applied by VI LCIA to metal samples for pitting corrosion determination, described on this document, the blob analysis of IMAQ library from LabVIEW 2010 [17] was applied. This technique involves several vision operations and analytical procedures, which detect within the image any 2D object which could be a potential pit located on the studied corroded metal surface. The image processing generates blobs and then works with them to calculate the area and perimeter of the pit, and the number of the generated blobs is an important characteristic for pits distinguishing. Blob (binary large object) is a group of interconnected pixels that have the same logic state and the same light intensity [18].

The basic structure of an image recognition VI by the application of blob analysis in IMAQ LabVIEW consists of image acquisition, histogram calculation, thresholding, and blob's filtering and analysis.

**Figure 6:**
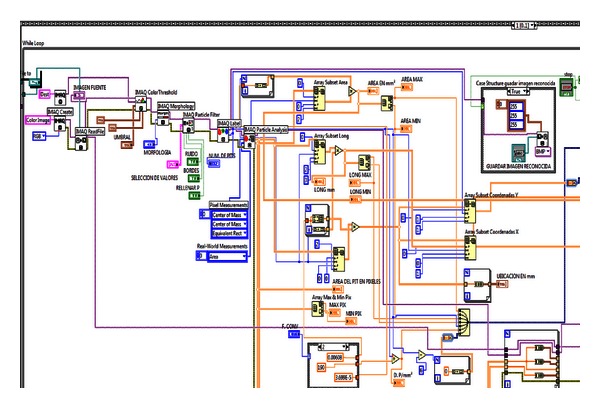
Blobs analysis and pixel conversion to metric units.

The image recognition VI for pitting corrosion application has all these components as well as the morphology and particle labeling to improve the shape and definition of pits under investigation. The programming diagram of the image analysis sub-VI is shown in Figure 6.

By means of the icons IMAQ create and IMAQ read file the precaptured images are acquired into the sub-VI for digitizing. After that, the color threshold process is performed to turn the image into binary format in the first section. Once the threshold process is done, the desired morphology of pitting is predefined by IMAQ morphology, and then a filtering process is performed by IMAQ particle filter which can be used to clean up the image deleting the nondesired particles defined as noise. The pitting spots in the already clean image are labeled and highlighted in different colors, by IMAQ label, so they can be easily distinguished. Finally, the pitting spots which are pixels are counted to be converted in real metric units by IMAQ particle analysis, and saved together with the LCIA generated parameters as: pit location, area, and transverse length.

Once the blob analysis is performed, the system proceeds to convert pixels to metric units for all the parameters determined by the blob analysis (program process shown in Figure 5). The conversion to square millimeters of the area is performed by the subarray from *array subset area*; the conversion to millimeters of the length is performed by the subarray from *array subset length,* and finally the location by coordinates in the subarrays (X, Y) performed by *array subset for coordinate X and coordinate Y*, respectively.

## 4. Applications

**Figure 7:**
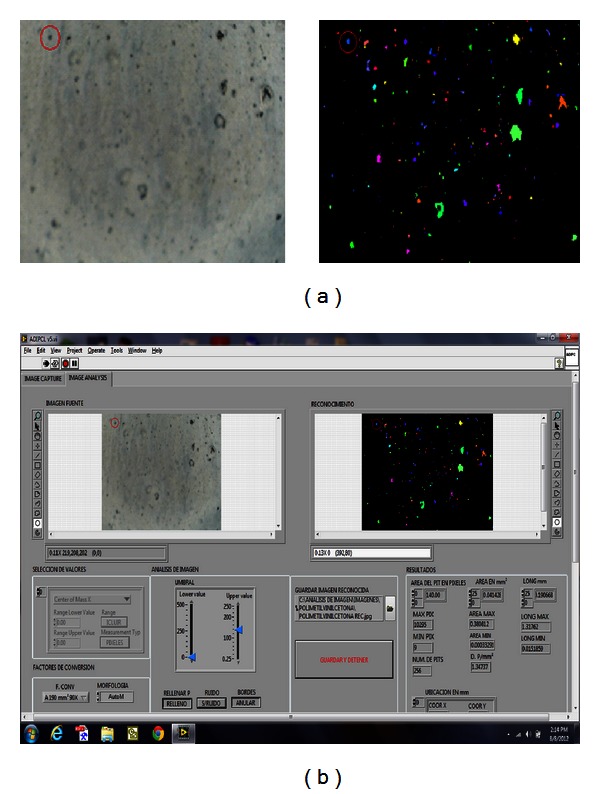
(a) Left: specimen microscope image; (a) right: map of the probable pits areas created by LCIA using the microscope image; (b) the LCIA user interface.

The system VI LCIA was applied for image recognition in localized corrosion studies of aluminum alloys AA6061-T3 specimens determining the number, density, and coordinates of probable pits areas together with their dimensions. Then, the created database file was employed for real-time scanning of the affected by the corrosion areas only employing SVET improving thus the scanning time more than ten times compared with the time for complete SVET scan of the entire surface. In Figure 7(a) left is shown the microscope image of a carbon steel UNS G10180 sample obtained with VI LCIA application. There, one of the pits is highlighted with a circle as an example, and in the right part of the same figure its identification by VI LCIA is shown determining its area to be 0.041 mm² and its transversal length to be 1.76 × 0.94 mm as displayed in the graphic interface of the image analysis sub-VI. These values fit very well with those obtained by the SVET device and the AMF microscope application. The total time for the SVET scan of the 25 × 25 mm specimen with the LCIA application was found to be about 4% only of the total time required for complete SVET scan of the same specimen achieving an acceleration of about 25 times [14].

## 5. Conclusions

A virtual instrumentation system called VI LCIA based on LabVIEW 2010 was developed allowing rapid determination of the pits number on large metal specimens. The VI LCIA controls synchronously the focus adjustment and the image taking by a digital microscope and the image analysis, resulting in detection and the mapping of the probable pits containing zones on the corroded metal specimen with their traverse length and density using blobs analysis. The created map is further used by a homemade SVET VI system with its own LabVIEW 2010 software to control a rapid performing (one pass scan) “true/false” check of the probable pits containing zones and also is able to perform a complete SVET scan of the already recognized “true” zones to determine the corrosion rate.
